# Circulating CTRP1 Levels in Type 2 Diabetes and Their Association with FGF21

**DOI:** 10.1155/2016/5479627

**Published:** 2016-05-23

**Authors:** Sora Han, Jong Dai Kim, Sunyi Lee, Ae Lee Jeong, Jeong Su Park, Hyo Jeong Yong, Ariundavaa Boldbaatar, Hye In Ka, Eun-Jung Rhee, Won-Young Lee, Young Yang

**Affiliations:** ^1^Department of Biological Sciences, Sookmyung Women's University, 04310 Seoul, Republic of Korea; ^2^Department of Internal Medicine, Konyang University Buyeo Hospital, Buyeo, Republic of Korea; ^3^Division of Endocrinology and Metabolism, Department of Internal Medicine, Sungkyunkwan University School of Medicine, Kangbuk Samsung Hospital, Seoul, Republic of Korea

## Abstract

The goal of this study was to investigate whether circulating C1q/TNF-*α*-related protein 1 (CTRP1) levels are associated with diabetes. In addition, relationships between CTRP1 and other diabetes-related cytokines were elucidated, including adiponectin and fibroblast growth factor 21 (FGF21). A total of 178 subjects (78 men and 100 women) aged 29–70 years (mean age, 46.1 years) were randomly selected. The sera from a normal glucose tolerance group (*n* = 68) and a prediabetes/type 2 diabetes group (*n* = 110) were collected; then, circulating levels of CTRP1, adiponectin, and FGF21 were determined via enzyme-linked immunosorbent assay in all sera. Subjects with either prediabetes or diabetes exhibited higher circulating CTRP1 levels than healthy subjects. Sera analysis revealed that CTRP1 was positively correlated with age, body mass index, fasting blood glucose, and circulating FGF21 levels. However, CTRP1 was negatively correlated with total cholesterol and total circulating adiponectin levels in univariate analysis. In addition, multivariate analysis found that CTRP1 was independently associated with age, fasting blood glucose, and circulating FGF21 levels. CTRP1 was correlated with homeostasis model assessment-*β* (HOMA-*β*), but no correlation was observed with HOMA-insulin resistance. In conclusion, circulating CTRP1 levels are increased in subjects with type 2 diabetes and are positively associated with circulating FGF21 levels.

## 1. Introduction

Glucose metabolism is systemically regulated by the endocrine system. In addition to insulin and glucagon, a variety of secreted proteins including adiponectin, fibroblast growth factor 21 (FGF21), and C1q/TNF-*α*-related protein 1 (CTRP1) influence each other to regulate glucose homeostasis. Abnormal production and secretion of those hormones result in defects in glucose metabolism by causing insulin resistance (IR) in the livers, skeletal muscles, and adipose tissues, which leads to type 2 diabetes mellitus (T2DM) [1–3]. Thus, controlling the concentration or activity of these newly elucidated hormones is regarded as an alternative therapeutic approach to improve insulin sensitivity in T2DM [4–6].

CTRP1 has recently been found to be a novel metabolic hormone [7]. CTRP1 shares its structural homology with adiponectin [8] and has been identified as a novel adipokine that is ubiquitously expressed, unlike adiponectin [7, 9]. Although the specific CTRP1 receptor has not yet been elucidated, studies with rodents show that administration of CTRP1 is sufficient to lower blood glucose levels and increase energy expenditure by activating the AMPK signaling cascade in the skeletal muscles [10]. Clinical studies show that circulating CTRP1 levels are positively correlated with fasting blood glucose levels and homeostatic model assessment-insulin resistance (HOMA-IR) in Chinese patients and nonalcoholic fatty liver patients [11–14]. It was also reported that circulating CTRP1 levels are higher in subjects with metabolic syndrome than those in healthy subjects [15]. However, it remains unclear whether the functional role of increased CTRP1 is to enhance or to aggravate insulin sensitivity, although CTRP1 is known to increase fatty acid oxidation and oxygen consumption in animal studies [12, 14, 16].

In this clinical study, circulating CTRP1 levels were measured in Korean subjects with normal glucose tolerance, prediabetes (preDM), and T2DM. Furthermore, levels of circulating adiponectin and FGF21 which are responsible for maintaining glucose homeostasis were measured to assess the relationship between those secreted proteins and CTRP1.

## 2. Materials and Methods

### 2.1. Study Subject

Healthy subjects were recruited from a population of patients who visited the Health Screening Center at Kangbuk Samsung Hospital in 2003. In total, 178 subjects who participated in an annual health checkup were randomly selected for this study. Subjects with viral hepatitis, other liver diseases, chronic renal disease, hypertension, excessive alcohol consumption (>20 g/day), or fasting blood glucose higher than 100 mg/dL were excluded [17]. Subjects with preDM consisted of the Kangbuk Samsung Hospital-Diabetes Prevention Project cohort, who had undergone comprehensive health examination and subsequently underwent an oral glucose tolerance test (OGTT). These subjects agreed to join a cohort study at the Kangbuk Samsung Hospital from December 2011 to July 2013. After a routine health examination, subjects who had shown a fasting blood glucose equal to or greater than 100 mg/dL were referred to the outpatient clinic in the endocrinology department. Then, they underwent a standard 2 h 75 g OGTT. PreDM was diagnosed when fasting blood glucose levels were between 100 and 126 mg/dL and 2 h levels were between 140 and 200 mg/dL. T2DM was diagnosed when fasting blood glucose levels were higher than 126 mg/dL or HbA1c was higher than 6.5%. This study protocol was approved by the Institutional Review Board and the Ethics Committee of Kangbuk Samsung Hospital and was carried out according to the principles of the 1975 Declaration of Helsinki. Written informed consent was provided by all subjects.

### 2.2. Laboratory Examinations

All sera were obtained in the morning, following 12–14 h of fasting. Sera were distributed to tubes and stored at −80°C for further experiments. Circulating levels of fatty acid-binding protein 4 (FABP4), monocyte chemoattractant protein 1 (MCP1), retinol-binding protein 4 (RBP4), interleukin-6 (IL-6), visfatin, tumor necrosis factor-*α* (TNF-*α*), CTRP1 (all from Biovendor, Brno, Czech Republic), FGF21, and total adiponectin (all from Alpco Diagnostics; Salem, NH, USA) were measured using an enzyme-linked immunosorbent assay (ELISA) according to the manufacturer's protocols. Blood glucose, total cholesterol, triglycerides (TGs), high-density lipoprotein cholesterol (HDL-C), and low-density lipoprotein cholesterol (LDL-C) were measured as previously described [18]. To estimate insulin sensitivity and *β*-cell function, HOMA was used, which is based on fasting blood glucose and insulin levels [19].

### 2.3. Statistical Analyses

All data are presented as the mean ± SD or proportion except where otherwise indicated. The following tests were used to compare the clinical and laboratory parameters according to circulating CTRP1 level tertile: one-way analysis of variance, Tukey's* post hoc* test, and the linear-by-linear association test. Pearson's and Kendall's tau-b correlation coefficients were calculated to evaluate the associations between circulating CTRP1 levels and clinical/laboratory measurements. Multiple regression analyses were performed to find an independent factor for CTRP1. Age, sex, body mass index (BMI), total cholesterol, systolic/diastolic blood pressure, fasting blood glucose, triglycerides, FGF21, adiponectin, and medications (sulphonylurea, DPP-IV inhibitor, alpha-glucosidase inhibitor, insulin, ACE inhibitor, ARB, thiazide, statin, fibrate, thioctic acid, and cilostazol) are included in this model and all variables controlled each other. All statistical analyses were performed using a SPSS version 18.0 (Chicago, IL, USA). *P* values < 0.05 were considered statistically significant.

## 3. Results

### 3.1. Baseline Characteristics of Subjects with PreDM and T2DM

The average age of the 178 individual study subjects was 46.1 ± 12.0 years, and their average BMI was 23.9 ± 3.1 kg/m^2^. Study subjects were categorized as healthy, preDM, or T2DM, according to fasting blood glucose level, OGTT, and HbA1c level. The levels of fasting blood glucose, insulin, HbA1c, and circulating FGF21 were significantly increased in subjects with preDM and T2DM, compared to healthy subjects. However, unlike other parameters, circulating adiponectin levels were negatively correlated with the fasting blood glucose level (Table 1).

### 3.2. Relationship between Circulating CTRP1 Levels and T2DM

**Figure 1:**
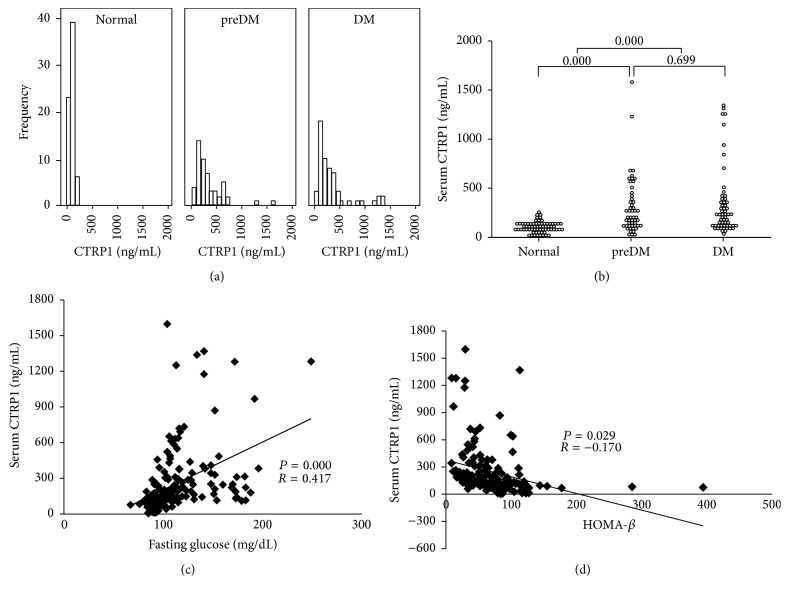
Circulating CTRP1 levels in healthy subjects and subjects with DM. Circulating CTRP1 levels were measured in three groups: normal, preDM, and DM patients. (a) Frequencies and distributions of circulating CTRP1 levels in three groups. (b) Circulating CTRP1 levels and statistical significance within three groups. Correlation analyses between circulating CTRP1 and fasting blood glucose levels (c) and HOMA-*β* (d). preDM, prediabetes mellitus; DM, diabetes mellitus; HOMA-*β*, homeostasis model assessment-*β*.

It was observed that the population showing high circulating CTRP1 level (over 300 ng/mL) was more distributed in preDM or T2DM groups than normal group (Figure 1(a)). Additionally, circulating CTRP1 levels were significantly increased in both subjects with preDM (352.8 ± 327.9 ng/mL) and T2DM (355.3 ± 341.3 ng/mL), compared with normal subjects (103.7 ± 53.4 ng/mL) on average. However, there was no statistical significance between circulating CTRP1 levels in subjects with preDM and T2DM (Figure 1(b)). As expected and previously reported [12, 14], circulating CTRP1 levels were positively correlated with fasting blood glucose levels (Figure 1(c)). Even when BMI, age, sex, and medications were controlled, CTRP1 independently and positively correlated with fasting blood glucose levels (Table 2). On the other hand, circulating CTRP1 levels were significantly and negatively correlated with HOMA-*β* (Figure 1(d)), while there was no correlation between CTRP1 and HOMA-IR (Table 3).

### 3.3. The Association of CTRP1 with Other Clinical and Laboratory Parameters

**Figure 2:**
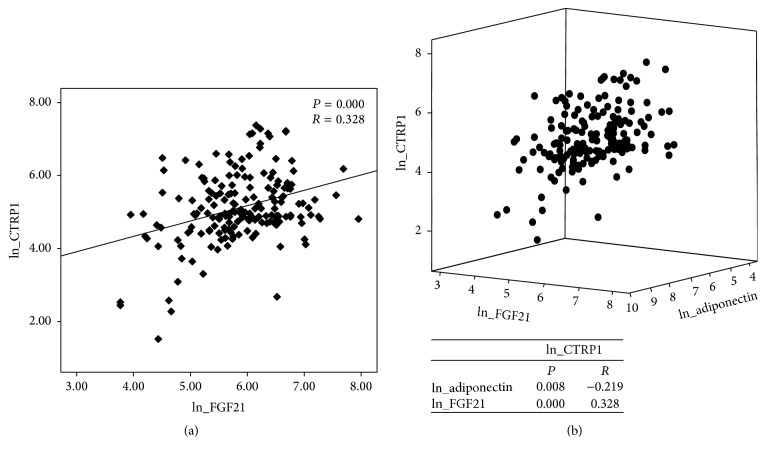
Correlation between circulating CTRP1 and DM-related cytokines. Circulating levels of CTRP1, FGF21, and adiponectin were measured in human sera. (a) Correlation analyses between circulating levels of CTRP1 and FGF21 in sera. (b) Correlation analyses between circulating levels of CTRP1, FGF21, and adiponectin in sera are showed in 3-dimensional dot plot (upper) and correlation coefficient (bottom). Correlations between indicated groups were analyzed using a generalized linear model. ln, natural logarithm.

Next, Pearson's correlation analysis was performed to assess the relationship between CTRP1 and other clinical and laboratory parameters in study subjects. As shown in Table 4, circulating CTRP1 levels were positively correlated with BMI, fasting blood glucose, and TG levels, whereas they were negatively correlated with total cholesterol and circulating adiponectin levels in univariate analysis. Interestingly, it was revealed that circulating CTRP1 levels were positively correlated with circulating FGF21 levels. Multivariate analysis revealed that fasting blood glucose and TG levels showed an independent relationship with circulating CTRP1 levels. Circulating FGF21 and adiponectin levels also showed a strong correlation with circulating CTRP1 levels (Table 5). Furthermore, when the parameters were additionally adjusted for age, sex, BMI, and medications, circulating CTRP1 levels were negatively correlated with circulating adiponectin levels and positively correlated with circulating FGF21 levels (Table 6, Figure 2(a)), whereas circulating FGF21 and adiponectin levels showed a negative correlation (Figure 2(b)). Because CTRP1 production is increased by TNF-*α* and IL-1*β* in adipose tissue [8, 20], we next assessed the relationship between circulating CTRP1 and other inflammatory cytokines (Table 7). Unexpectedly, no significant correlation was observed between circulating CTRP1 levels and other inflammatory cytokines.

## 4. Discussion

Previously, Xin et al. showed that circulating CTRP1 levels are elevated in Chinese subjects with T2DM and independently correlated with T2DM risk factors including BMI, fasting blood glucose levels, HbA1c, LDL cholesterol, and TNF-*α* [12]. In this study, circulating CTRP1 levels were positively and independently correlated with circulating FGF21 levels as well as BMI and fasting blood glucose levels in Korean subjects. In addition, we also show that circulating CTRP1 levels were significantly increased in subjects with preDM compared to healthy subjects, suggesting that CTRP1 is a prospective marker to prediagnose T2DM. Therefore, further investigation is necessary to reveal how CTRP1 affects impairment of glucose metabolism in the early stages of DM. At least, CTRP1 may affect insulin secretion because circulating CTRP1 levels were significantly associated with HOMA-*β*.

The previous studies reported that the administration of recombinant CTRP1 decreases blood glucose levels through activating the AMPK pathway and facilitating glucose uptake in the skeletal muscles in mice and that CTRP1 transgenic mice show an improved metabolic index against a high-fat diet [10]. Regardless of these beneficial effects of CTRP1 on glucose metabolism, subjects with T2DM show increased circulating CTRP1 levels compared to healthy subjects. Similar to CTRP1, circulating FGF21 levels in subjects with T2DM were also unexpectedly higher compared to those of healthy subjects, although the administration of recombinant FGF21 in obese and diabetic rodent models ameliorates hyperglycemia and dyslipidemia by the enhancement of insulin sensitivity and glucose uptake [21]. These paradoxical results can be explained by the fact that a hyperglycemic state increases the compensatory secretion of CTRP1 and FGF21 [12]. However, the increased CTRP1 and FGF21 in subjects with T2DM fail to restore glucose metabolism. This can be explained by a possibility that abnormal physiological condition in T2DM may diminish the abilities of CTRP1 and FGF21 to enhance glucose metabolism. There is a report to support this possibility. Zhang et al. showed that metabolic imbalance in T2DM causes the FGF21 resistance [22]. Thus, whether CTRP1 also shows resistance in T2DM may be worth studying.

In the point of view that both circulating CTRP1 and FGF21 levels are increased in subjects with T2DM, the functional relationship between CTRP1 and FGF21 would be expected. CTRP1 and FGF21 have similar beneficial effects on glucose metabolism and they also share extracellular signal-regulated kinase 1/2 (ERK1/2) and AMPK as downstream signal molecules [10, 23–25]. Thus, it is possible that CTRP1 and FGF21 are related with each other in balancing glucose homeostasis. However, further functional studies should be conducted to determine whether the relationship between CTRP1 and FGF21 is parallel, vertical, or synergistic in glucose metabolism.

There were some limitations in our study. First, we did not perform OGTT in healthy subjects. Although their fasting blood glucose levels were less than 100 mg/dL, they could possibly be classified in the impaired glucose tolerance group if they performed accordingly on the OGTT. Second, we did not have HbA1c information from healthy subjects because we did not acquire samples to evaluate this measure. Third, subjects with T2DM treated as patients at the clinic were taking antidiabetic medications. Although the adjustment for medications was performed, cytokine and HOMA values might already have been altered by the therapeutic effect.

In conclusion, this study shows the relationship between CTRP1 and FGF21. Further studies will shed light on how CTRP1 and/or FGF21 affect physiological function in subjects with T2DM.

## Figures and Tables

**Table 1:** Baseline characteristics of study participants.

	Normal (*n* = 68)	PreDM (*n* = 52)	DM (*n* = 58)	*P* value (normal versus PreDM)	*P* value (PreDM versus DM)
BMI (kg/m^2^)	22.45 ± 2.7	24.28 ± 2.9	25.3 ± 3.1	0.001	0.097
SBP (mmHg)	106.6 ± 11.4	115.2 ± 16.0	117.9 ± 11.1	0.002	0.410
Glucose (mg/dL)	90.0 ± 5.5	108.8 ± 6.3	141.8 ± 32.1	0.000	0.000
Insulin (mg/dL)	7.4 ± 2.1	10.0 ± 4.9	12.0 ± 7.2	0.000	0.083
HOMA-IR	1.6 ± 0.5	2.7 ± 1.5	4.4 ± 3.3	0.000	0.000
HOMR-*β*	103.6 ± 37.0	79.3 ± 35.3	60.2 ± 32.7	0.000	0.011
HbA1c (%)	No data	5.8 ± 0.3	7.2 ± 0.9	NA	0.000
Adiponectin (ng/mL)	4427.0 ± 2043.6	2760.7 ± 2112.3	1483.3 ± 1135.6	0.000	0.001
FGF21 (pg/mL)	379.2 ± 327.4	502.8 ± 425.1	546.8 ± 385.6	0.026	0.766

All data are presented as the mean ± SD.

PreDM, prediabetes patient; DM, diabetes patient; BMI, body mass index; SBP, systolic blood pressure; HOMA-*β*, homeostasis model assessment-*β* cell function; HOMA-IR, homeostasis model-insulin resistance; HbA1c, glycated hemoglobin; NA, not applicable.

**Table 2:** Correlation between circulating CTRP1 and fasting blood glucose levels.

	CTRP1^†^	
	Coefficient	*P* value
Glucose	0.310	0.000

^†^Ln transformed. BMI, age, sex, and medications were controlled variables and medications included are SU, glinide, AGI, DPP-IV inhibitor, insulin, ACE inhibitor, ARB, CCB, thiazide, *β*-blocker, statin, fenofibrate, cilostazol, and thioctic acid.

**Table 3:** Pearson's correlation coefficient of CTRP1 with HOMA-*β* and HOMA-IR.

	CTRP1	
	Coefficient	*P* value
HOMA-*β*	−0.170	0.029
HOMA-IR	0.030	0.706

HOMA-*β*, homeostasis model assessment-*β* cell function; HOMA-IR, homeostasis model-insulin resistance.

**Table 4:** Pearson's correlation coefficient between circulating CTRP1 levels and clinical data in univariate analysis.

	Coefficient	*P* value
Sex	NA	0.904
BMI	0.196	0.009
SBP	0.125	0.145
DBP	0.154	0.072
Glucose	0.455	0.000
Total cholesterol	−0.180	0.018
HDL cholesterol	−0.124	0.106
LDL cholesterol	−0.145	0.061
TGs	0.167	0.030
Adiponectin	−0.365	0.000
FGF21	0.341	0.000

BMI, body mass index; SBP, systolic blood pressure; DBP, diastolic blood pressure; HDL, high-density lipoprotein; LDL, low-density lipoprotein; TGs, triglyceride.

**Table 5:** Correlation between circulating CTRP1 levels and clinical data by multivariate analysis.

	Unstandardized *β* ± SE	Standardized *β* coefficient	*P* value
Sex	−0.008 ± 0.157	−0.012	1.000
BMI	−0.014 ± 0.028	−0.046	0.626
SBP^†^	−0.266 ± 0.685	−0.034	0.699
Glucose^†^	1.826 ± 0.604	0.420	0.003
Total cholesterol	0.002 ± 0.002	0.101	0.310
TGs^†^	−0.301 ± 0.166	−0.174	0.073
Adiponectin^†^	−0.089 ± 0.114	−0.081	0.435
FGF21^†^	0.309 ± 0.094	0.261	0.001
Medication			Each variable is NS
Model	*R* ^2^ (0.423)		0.001

^†^Ln transformed. Medications included are SU, glinide, AGI, DPP-IV inhibitor, insulin, ACE inhibitor, ARB, CCB, thiazide, *β*-blocker, statin, fenofibrate, cilostazol, and thioctic acid. SE, standard error; BMI, body mass index; SBP, systolic blood pressure; TGs, triglycerides; NS, not significant.

**Table 6:** Kendall's tau-b correlation between circulating levels of CTRP1, adiponectin, and FGF21.

	Adiponectin^†^	FGF21^†^
Correlation coefficient	−0.219	0.328
*P* value	0.008	0.000

^†^Ln transformed. Medications included are SU, glinide, AGI, DPP-IV inhibitor, insulin, ACE inhibitor, ARB, CCB, thiazide, *β*-blocker, statin, fenofibrate, cilostazol, and thioctic acid.

**Table 7:** Kendall's tau-b correlation between circulating levels of CTRP1 and other cytokines under normal conditions.

	FGF21	RBP4	IL-6	MCP1	TNF-*α*	Visfatin	AFABP
Correlation coefficient	0.199	−0.026	−0.059	0.051	−0.028	−0.012	0.037
*P* value	0.017	0.755	0.478	0.539	0.744	0.896	0.660

FGF21, fibroblast growth factor 21; RBP4, retinol-binding protein 4; IL-6, interleukin-6; MCP1, monocyte chemoattractant protein 1; TNF-*α*, tumor necrosis factor-*α*; AFABP, adipocyte fatty acid-binding protein.
